# Integrating machine learning, deep learning, and image analysis for seed species classification

**DOI:** 10.1002/aps3.70072

**Published:** 2026-07-23

**Authors:** Jonathan Ashworth, Eloise Barrett, Rebonto Haque, Ben Wildey, Anthony Hall, Felix Shaw, Jose J. De Vega

**Affiliations:** ^1^ Earlham Institute Norwich Research Park Norwich NR4 7UZ United Kingdom

**Keywords:** habitat restoration, image analysis, machine learning, seed provenance, species classification, wildflowers

## Abstract

**Premise:**

The growing demand for wildflower seeds in ecological restoration requires reliable species identification, yet current market products often contain heterogeneous species. As seed identification is labor‐intensive and requires advanced botanical knowledge, we evaluated multiple segmentation and classification approaches to determine which combination performed better for identifying species and quantifying their frequencies under real‐life constraints.

**Methods:**

We generated seed images from common wildflower species using a flatbed scanner. Artificial intelligence (AI) and non‐AI segmentation pipelines were compared. We evaluated over 20 classifiers on tabulated features and convolutional neural networks (CNNs) on single seed image inputs. Open‐set conditions were simulated using “mock seed mixes” with previously unseen taxa using decision thresholds.

**Results:**

With tabulated data, the XGBoost and Multi‐layer Perceptron (MLP) models exceeded F1 > 0.96, while AutoGluon's CNN and ensemble models reached F1 > 0.97. ResNet‐50 achieved an F1 score over 0.99 on single‐seed images obtained with Cellpose segmentation. Random forest achieved the highest accuracy (0.92) with unseen species in open‐set classification.

**Discussion:**

Deep learning segmentation substantially enhanced CNN accuracy, yet tabulated‐feature machine learning models remained competitive and efficient. Threshold‐based rejection enabled robust open‐set classification, with random forest outperforming deeper models in rejecting unseen seeds. No single method was universally optimal; the best strategy depends on whether the task involves closed‐set or open‐set classification and on trade‐offs between accuracy and computational cost.

There is a growing and sustained demand for seeds of native plant species for habitat creation and enhancement (Walker et al., 2004; MacIntyre, 2024), prompting increased cultivation to meet this market demand. Most wildflower seeds on the market derive from farming cultivation, because wild collection alone cannot come close to meeting the scale and seasonality of demand (MacIntyre, 2024). This highlights that supply chains for native seed components are critical factors for restoration success and the need for quality assurance within them.

Cultivation can alter the genetic and phenotypic composition of the seed stock across generations. As sources of wild material are limited (founder effect) and the cultivation process is vulnerable to unintended selection (Conrady et al., 2022), monitoring and controlling the genetic and phenotypic diversity of native flower seeds is essential to ensure the ecological value of these reintroduced species and to sustain the fitness of the newly created habitats in the long term (Basey et al., 2015; Espeland et al., 2017). Therefore, practical monitoring methods are needed that scale with commercial needs while remaining accessible to non‐specialists.

Here, we aim to create a cost‐effective and accessible pipeline for identifying wildflower species and quantifying them in seed mixes. We plan to use this method for our own research, but also believe that an easy‐to‐use seed classifier would benefit anyone involved in the value chain of native seed products. Identification of seeds to species level using morphological characters (e.g., color, shape, size) is a specialist skill (ISTA, 2025), which can limit stakeholder access to critical information. At the same time, the native seed market is often inadequately regulated compared to the arable equivalent (Durka et al., 2025). In the absence of regulatory guidance, native seed suppliers have adopted a broad range of strategies for the production and marketing of seed products, including around composition (selection and proportions of species) and labeling (Walker et al., 2004; Barry and Hodge, 2023; MacIntyre, 2024).

Although a few species occur frequently in mixed products, possibly due to their ease of cultivation, a wide range of species can be included in commercial seed mixes. This challenge should be considered when designing a classification strategy, as it will lead to open‐set classification scenarios. This is likely due to multiple factors affecting immediate availability to producers (supply), as well as the demands of high‐volume customers and the incentive structure introduced by land stewardship regulations, which are often the source of the funds for these interventions. For example, in the United Kingdom, the statutory biodiversity metric (BM) and UK Habitat Classification (UKHab) can incentivize the presence of particular wildflower species over others (UKHab Ltd., 2023). Closely related species and agricultural varieties of the same species represent a particular challenge for practitioners. As a result, a monitoring system must be able to discriminate among closely related taxa and handle previously unseen species.

Previous image‐based seed classifiers have adopted a range of methods for data acquisition (imaging setup), feature selection, and statistical model architecture (Ahmad et al., 1999; Granitto et al., 2002; Grillo et al., 2010; Zhao et al., 2022; Steinecke et al., 2023; Reek et al., 2024). For image acquisition, early approaches relied heavily on handcrafted morphological and color descriptors extracted from controlled imaging environments. For example, Ahmad et al. (1999) used red‐green‐blue (RGB) color ratios and simple shape metrics to differentiate healthy from diseased soybean seeds using linear discriminant analysis, achieving accuracies around 90%. More recent work has incorporated larger feature sets and ensemble learners: Reek et al. (2024) applied random forests to 25–30 handcrafted morphological traits and achieved ~80–95% accuracy depending on species set and imaging conditions, while Steinecke et al. (2023) combined segmentation with automated measurement of size and texture traits to enable trait‐based identification in small‐seeded species. Computer vision deep learning models have been predominantly crop‐focused and successfully identified seed species or varieties (Zhao et al., 2022). Khatri et al. (2022) and Madhavan et al. (2023) used convolutional neural networks (CNNs) to classify maize and wheat varieties, often achieving an F1 score over 90%, but within narrow taxonomic and phenotypic ranges. Reviews by Zhao et al. (2022) and Luo et al. (2023) highlight that performance drops markedly when species diversity increases or when training data come from heterogeneous provenances. Because prior studies focus on limited taxonomic scopes, controlled imaging, and small provenance variation, their reported high accuracies do not generalize to heterogeneous, multi‐species wildflower datasets or open‐set scenarios. These needs are not addressed by existing seed‐classification solutions. Therefore, we tested a broader suite of modern models to evaluate robustness, scalability, and transferability under realistic market conditions.

For image acquisition, we preferred a flatbed document scanner for its lower cost and user‐friendly design, which has been successfully applied in similar situations (Vale et al., 2020; Dayrell et al., 2023; Loddo et al., 2023). We preferred flatbed document scanners over alternatives such as a digital single‐lens reflex camera (DSLR) or mirrorless camera rigs, smartphone imaging, or custom macro‐photography boxes because scanners offer uniform, shadow‐free illumination, stable geometry, and ease of standardization across users. Unlike camera‐based setups, scanners reduce variation caused by lighting angle, distance, lens distortion, and operator handling, while remaining affordable and widely accessible. This makes scanners a preferable solution for data acquisition in our conditions.

In this study, we aim to identify the best approach to species identification for each seed in a mix under two conditions: (i) closed versus open‐set classification (given the sampling specifications introduced previously) and (ii) performance versus computational cost. We believe there is no one‐size‐fits‐all solution; instead, a combination of best practices should be tailored to each case. Consequently, we frame our evaluation around accuracy–cost trade‐offs and robustness to identification of unseen taxa.

To that end, we have developed two segmentation pipelines—one artificial intelligence (AI)‐based (Cellpose v4; Stringer et al., 2021) and another using classic image analysis techniques (ImageJ; Schneider et al., 2012)—to generate single‐seed masks for each seed starting from scanned images of seeds scattered across a domestic flatbed scanner. Then, we used an ImageJ plug‐in (Loddo et al., 2023) to extract numerical values for 64 features regarding the color, texture, and shape features of each seed. In parallel, we developed a Python script using OpenCV v2 (Bradski, 2000) to generate individual seed squared images from the masks to use with image‐based classification models. We obtained over 145,000 instances representing seeds from commonly traded wildflower species native to the United Kingdom, sourced from multiple origins or provenances. We trained and evaluated a wide variety of linear, ensemble, and neural network machine learning models on both tabular and image data. Because variation in seed morphology within a species arises from genetic and environmental factors, as well as seed production and management, we also evaluated and verified that the datasets accurately represented the overall phenotypic diversity of the species. Finally, we deployed these models to predict the species composition of “mock seed mixes” to simulate performance in real‐world scenarios, including unseen species in an open‐set classification context. Our findings provide practical guidance on when to use tabular or image‐based data, and whether machine learning or deep learning is more suitable.

## METHODS

### Image acquisition with a domestic flatbed scanner

Seeds of commonly traded wildflower species were purchased as single species (Appendix S1), from five separate suppliers each. Seeds of each species were handled individually and carefully checked for contamination. Seeds were scattered evenly over the bed of a domestic‐market Canon LiDE 300 A4 document scanner (Canon, Tokyo, Japan) at a resolution of 600 ppi, avoiding clusters of touching seeds (Figure 1). Masking tape was applied to the perimeter of the scanning surface to avoid seed ingress. In this way, image data for thousands of seeds can be rapidly captured in a single scan. All images were saved in a single directory in TIFF (.tif) image file format, labeled by species name.

**Figure 1:**
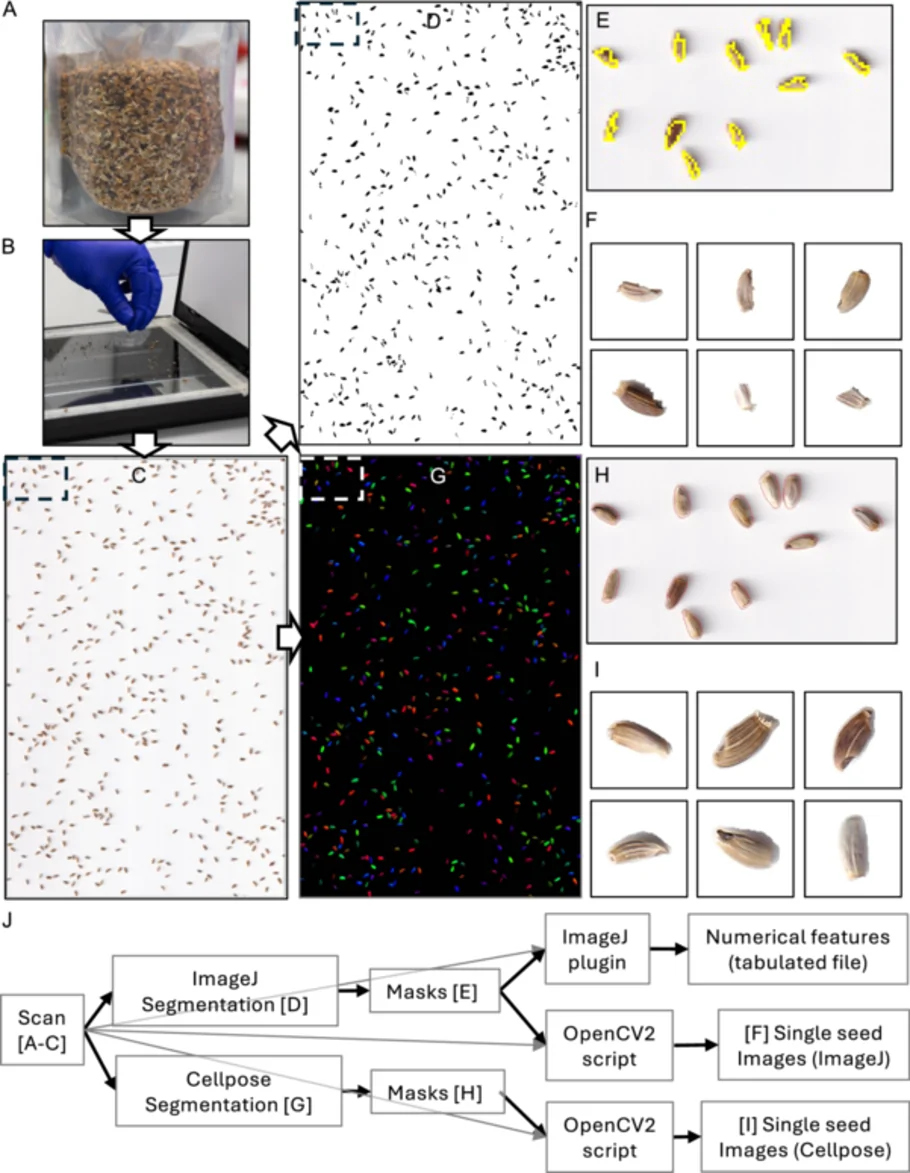
For image acquisition from seed mixes (A), seeds were scattered evenly on a domestic A4 document scanner bed (B, C). Two methods were used for image segmentation: a non‐AI ImageJ plug‐in and pipeline (D, E) was used for single seed image generation (F) and for the extraction of numerical features for each seed, and an AI‐based pipeline using the Cellpose library (G, H) was also used to capture single seed images (I).

### Image masking, segmentation, and numerical feature extraction with ImageJ

ImageJ (Schneider et al., 2012) was used for image processing tasks. We created a bespoke macro and modified a Java plug‐in to process and segment individual seeds from the scanned images. Our *Generic_Background_Analyzer* function is adapted from *SeedsAnalyser* (Loddo et al., 2023), which is an extension of the *ParticleAnalyzer* plug‐in in the main ImageJ program. The function relies on the *ThresholdAdjuster* function to identify the areas and objects in the image. The plug‐in version in Loddo et al. (2023) used a blue background to segment, which was not compatible with our image acquisition method. Our new version of the *Generic_Background_Analyzer* function uses the same *ParticleAnalyzer* subclass to extract features but also includes an additional algorithm to extract seeds from a neutral (white) background.

First, a macro crops the image to eliminate artifacts in the margins due to the taped edges. The plug‐in creates a duplicate of the image, while retaining the unmodified original image. The duplicate is then converted to an 8‐bit format to allow the use of predefined functions. The particle edges are located and used to create the first threshold, which produces a binary (black‐and‐white) image in which edges are dilated to eliminate “hole” artifacts caused by glare. Further dilation and erosion steps are applied to improve the accuracy of edge location, producing a clearer representation of the shape of each seed (i.e., a mask). Finally, this outline is superimposed onto the original image and acts as the region of interest (ROI) from which the features will be extracted.

In this study, we used 64 features: 32 morphological, 16 texture, and 16 color intensity–based (Appendix S2). Once all images are processed, the output is compiled into a single tabulated results table. To filter out seed clusters (groups of overlapping seeds recognized as a single seed) and dust and dirt particles, an *Outlier_Detector* R script was run to remove rows from the results table containing feature values three times greater than the upper and lower quartiles, respectively. The resulting filtered table was used for model training.

### Single‐seed image extraction with OpenCV and scikit‐image

We developed a Python module that automates the generation of individual seed images starting from the scanned seed images and their corresponding binary masks (generated in the previous section). The module identified distinct seeds within the mask using the *findContours()* function from OpenCV v2 (Bradski, 2000) and processed each individually. It then used the scikit‐image (van der Walt et al., 2014) function *morphology.label()* to identify groups of adjacent foreground pixels (“seed regions”) in the binary masks, and then measured properties with *measure.regionprops()* to iterate over each detected region, filter region outliers by size, and use the region's bounding box (region.bbox) to crop each seed from the original scan. Each seed is centered on the canvas and saved as an individual standardized square image (160 × 160 pixels) on a clean white background. For consistency, the script changes black pixels, which are artifacts of masking, to white pixels. All actions are logged (such as saved images, skipped regions, and errors) into a timestamped log file. This makes it suitable for batch processing and reproducible seed dataset creation from microscope or scanner images.

### Image masking and extraction with the Cellpose neural network

We implemented another automated pipeline to segment objects starting from the original scanner images using Cellpose v4 (v4‐compatible). Cellpose is a deep neural network–based tool designed for general‐purpose, cell‐level, and object‐level image segmentation in biological and microscopic images (Stringer et al., 2021), predicting object boundaries and shapes by learning spatial flow fields rather than relying solely on pixel classification.

Our pipeline accepts the scanned images and converts them to RGB values and then to normalized [0,1] arrays. The default pre‐trained “cellpose‐sam” model from Cellpose was used to predict segments and return an array of masks. We also tested the Omnipose model (Cutler et al., 2022) with the exact same results. The model output was post‐processed into TIFF images containing the masked seed scans. As before, scikit‐image and OpenCV were later used to generate individual square images of seeds from the original scanned images and their corresponding Cellpose masks, producing a cutout of the seed on a completely white background.

### Training and prediction with machine learning classification models from tabulated data

For tabulated data processing, dimension reduction (principal component analysis [PCA] and linear discriminant analysis [LDA]), and model training and evaluation, we primarily used the Python library Scikit‐learn (Pedregosa et al., 2011). XGBoost (Chen and Guestrin, 2016) was also included for its high performance on similar datasets (Sarijaloo et al., 2021; Huber et al., 2022). PCA used default parameters; LDA was performed using the default SVD solver and no shrinkage. Class priors were inferred from the data. Features were not standardized prior to LDA. We retained three linear discriminants for visualization and computed classification metrics to assess performance.

Our tabulated data were randomly split into 80% training and 20% test subsets (fivefold), using Scikit‐learn's *train_test_split* function with the stratify option to retain an equivalent proportion of species in the training and test datasets. The training dataset was balanced at the species level using random over‐sampling examples (ROSE) (Lunardon et al., 2014) from Scikit‐learn for all model training. F1 score, the harmonic mean of precision and recall, was selected as the primary metric for evaluating models. To establish the variance of the score, all training/evaluation cycles were repeated independently 50 times. Hyperparameter values were selected using GridSearchCV and optimized over a range of standard parameter values. Once the optimum hyperparameter combination was established from the cross‐validation, the final models were trained with the combined training and test datasets. These models were then saved and evaluated on a new evaluation set, which was held out of the cross‐validation process to eliminate the risk of data leakage. To explicitly test generalization across seed provenances (suppliers), we performed group‐wise splitting by supplier. For each model, we iteratively excluded one supplier from training (i.e., all instances from the held‐out supplier were removed from the training data), trained the model on the remaining suppliers as described above, and then evaluated the performance for each held‐out supplier. We report per‐class metrics for each evaluation (Appendix S3).

In addition to the use of base Scikit‐learn, AutoXAI4Omics v.1.0.1 (Strudwick et al., 2025), an explainable AutoML (Aragão et al., 2025) tool, was deployed to automatically build and compare automated model‐optimization features. This tool is based on Scikit‐learn algorithms, using *RandomOverSampler()* from Scikit‐learn, and also allowed us to test a neural network classifier based on AutoKeras (Jin et al., 2019). AutoGluon Tabular v1.1.1 (Erickson et al., 2020), another explainable AutoML software, was also tested to automate model building, optimization through hyperparameter‐tuning, and comparison across a broader range of algorithms. This tool includes complex ensemble and a variety of deep learning neural network architectures. AutoGluon implements an automated ensemble feature that determines the optimal stacking of divergent model types.

### Generation of datasets of open‐set “mock” seed mixes

To evaluate open‐set classification, a new collection of images from new species was created and never used in model training (held‐out) to ensure they were entirely novel to the models. These sets were also used to investigate the effect of the training size and diversity of seed sources (provenance); these data were also subsampled so that the dataset represents only materials from one, two, three, four, or five different suppliers. Each dataset was subsampled again to retain 200, 400, 600, 800, 1000, and 2000 instances (seeds) per species.

### Deep learning model training and evaluation from individual seed images

We implemented CNNs to classify seed images using three pretrained models (ResNet‐50 [He et al., 2016], InceptionV3 [Szegedy et al., 2016], and MobileNetV3 [Howard et al., 2019]) available in PyTorch v2.9.0. The workflow was developed in Python using PyTorch v2.9.0 for model and data handling and Scikit‐learn for metric reporting, with only the model and weights changed. The models were fine‐tuned for our dataset during training by optimizing the last layer using the AdamW optimization algorithm (Loshchilov and Hutter, 2017) with cross‐entropy loss over 100 epochs. This training used 700 images per class, split 80:20 between the training and validation subsets.

We decided to proceed with ResNet‐50 only for further optimization; each individual seed image was first standardized using the preprocessing pipeline recommended for the ResNet‐50 model, including color normalization and random geometric transformations to improve generalization. Images were resized and center‐cropped to the input dimensions expected by the network. Augmentations such as for random cropping, horizontal flipping, and color jittering were applied during training to reduce overfitting. To address uneven representation among taxa, class balancing was applied during both training and validation, giving additional weight to classes (species) with fewer seed images and preventing the network from being biased toward more abundant classes, using the PyTorch *WeightedRandomSampler* function. Model training was carried out for 100 epochs using the AdamW optimization algorithm with cross‐entropy loss. During training, only the final fully connected layer of the ResNet‐50 network was updated initially, with deeper convolutional layers progressively unfrozen in later epochs to allow fine‐tuning once the classifier stabilized. Model performance was monitored each epoch based on overall loss, accuracy, and macro‐F1 scores. These metrics were recorded for both training and validation and were plotted as learning curves to assess convergence and potential overfitting.

To evaluate model performance under more realistic conditions where some taxa may be absent from the training data (open‐set classification), the “new species” were deliberately held out from the training but introduced during validation. The validation set was evaluated under a range of probability thresholds (from 0 to 1 in increments of 0.02 or 0.05) to explore how the confidence to classify into each species (class) affected precision, recall, and F1 score. For each threshold value, predictions with a maximum class probability below the threshold were considered unclassified, whereas those above the threshold were assigned to the class of highest probability. We also monitored coverage, defined as the fraction of validation images that received a species classification (i.e., a probability above the given threshold). We implemented a second version of this script that retained the same preprocessing, data balancing, and model architecture described earlier. However, it incorporated a “decision threshold” variable, so the class with the maximum probability was taken as the predicted species only if its confidence exceeded the predefined decision threshold; otherwise, the image was categorized as “unclassified.”

## RESULTS

### Extraction of visual features in a collection of species‐labeled wildflower seeds

Seeds from the 10 wildflower species most frequently traded in the United Kingdom (Appendix S1) were purchased from five commercial suppliers. We generated 147 single‐species images scanned at 600 ppi, containing 145,771 individual seeds. Using a modified image segmentation pipeline in ImageJ, we extracted 64 numerical features related to each seed's shape, color, size, or texture, tabulated in a text file (Appendix S2). We generated enough scanned images for 2000 seeds per species per provider except in a few cases where it was not possible (Appendix S1) due to cost and availability.

To assess the discriminating power of the extracted numerical features, we performed dimension reduction using the full dataset. PCA has no concept of classes, so post‐hoc labeling enables us to visualize clustering by species. We retained three principal components for visualization (Figure 2A). Total variance explained (PC1 to PC3) was 62% (PC1: 32.59%, PC2: 21.79%, PC3: 7.62%). Principal component loadings segregated based on feature type; the top five features for each principal component are morphological (PC1), color (PC2), and texture (PC3) (Appendix S4). LDA revealed marked clustering at the species level, as well as improved variance representation on the first three components (Figure 2B; LD1: 38.95%, LD2: 23.80%, LD3: 11.99%), and the total variance explained (LD1 to LD3) was 74.74%. Without prior class balancing, parameter tuning, or feature standardization, LDA's internal cross‐validation provided a macro‐F1 score of 0.92.

**Figure 2:**
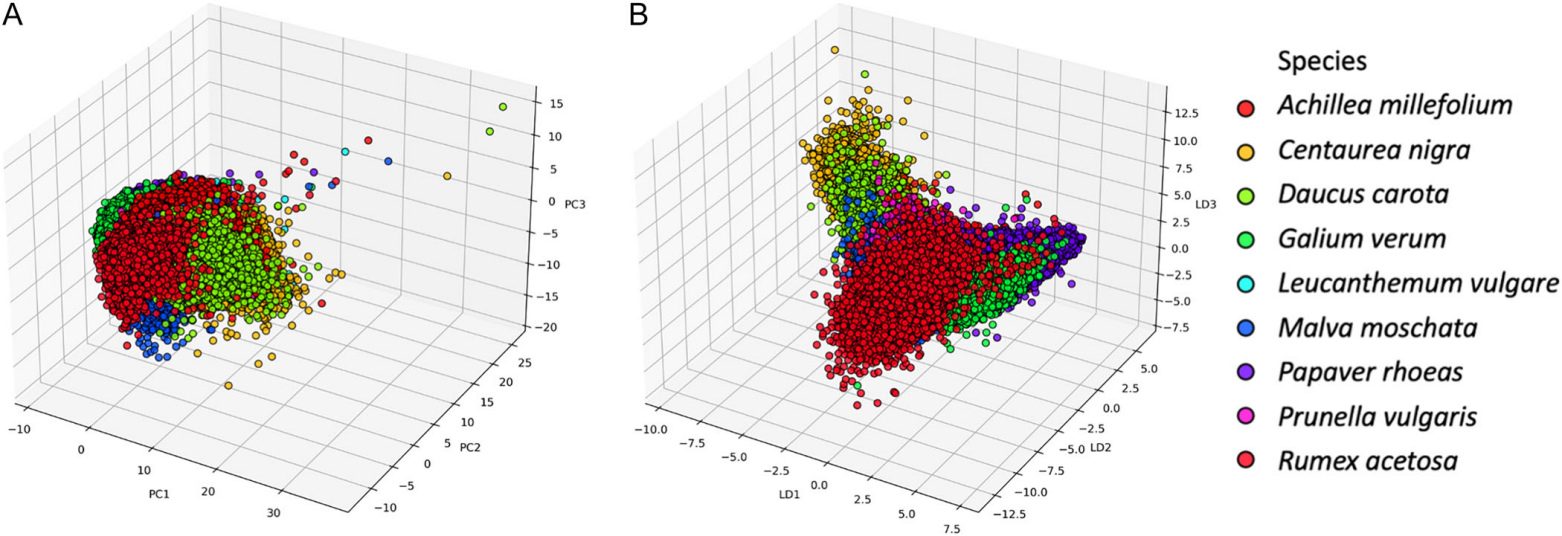
Comparison of species distribution based on extracted features. (A) A principal component analysis (PCA) 3D plot representing 64 features across 145,771 instances (seeds). (B) A linear discriminant analysis (LDA) 3D plot with the same features and samples using species as class entry.

### Validating dataset size and representativeness

Seeds were sourced from five commercial providers for each species. To validate the number of provenances used and the generated dataset size, we completed a fivefold cross‐validation experiment using XGBoost with datasets from one to five provenances (number of providers or suppliers) and training subset sizes ranging from 200 to 2000 instances (seeds). Across species, the impact on performance was measured by the mean macro‐F1 score (Figure 3).

**Figure 3:**
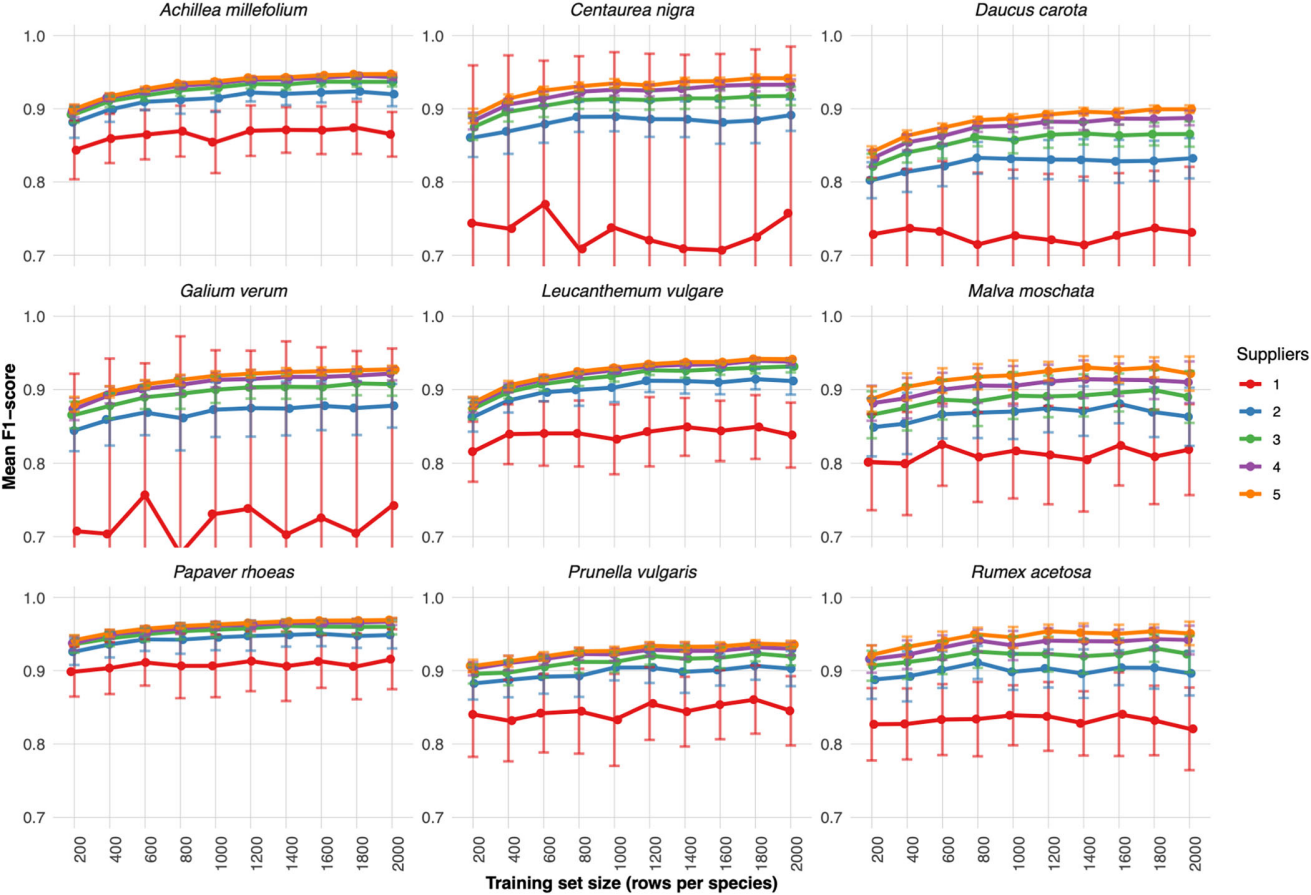
Macro‐F1 scores for the prediction of nine species from fivefold repeated cross‐validations using one to five provenances (represented by different color lines) and increasing number of instances (seeds) in the training dataset (*x*‐axis). The F1 score improved with an increasing number of providers and instances until it plateaued. Error bars for *Centaurea nigra*, *Daucus carota*, and *Galium verum* exceeded the range covered by the plot.

The mean F1 score was positively correlated with both training set size and number of sources (Figure 3). However, diminishing returns were obtained when more than three sources were sampled, and scores plateaued at the larger training subset sizes, generally over 1000 instances per species. The dataset generated from all five provenances was therefore considered to be of sufficient size and diversity, and was pooled for subsequent analyses.

### Comparison of machine learning and deep learning classification models from tabulated inputs

We assessed 20 classification models from the Scikit‐learn and AutoGluon machine learning libraries (Pedregosa et al., 2011; Erickson et al., 2020). The Scikit‐learn models were optimized using either the GridSearchCV function or the AutoXAI4Omics tool (Strudwick et al., 2025). AutoGluon offers additional deep learning and ensemble models not available from Scikit‐learn and automatically performs feature engineering and optimization. We trained all models on 80% of the dataset (training set; 116,617 instances) and tested them on the remaining 20% (evaluation set; 29,154 instances).

From Scikit‐learn, the Multi‐layer Perceptron (MLP) and XGBoost models marginally outperformed gradient boosting, histogram‐based gradient boosting (HGB), and random forest models, with mean F1 scores over 96%. The highest‐performing models from and optimized using AutoGluon were a FastAI neural network and bagged ensembles, consisting of the top‐performing models stacked together, named “ensembleL2” and “ensembleL3” (Figure 4, Table 1).

**Figure 4:**
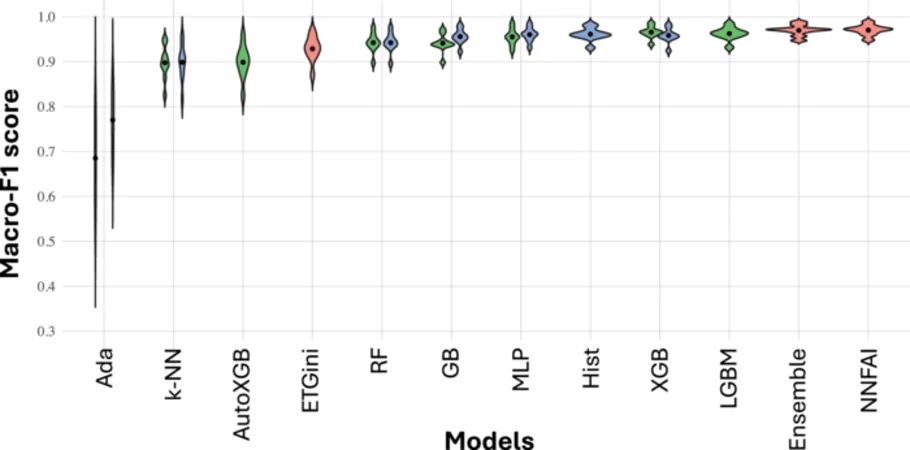
Macro‐F1 scores for species prediction performance for the 12 best‐performing machine learning models trained on tabulated features extracted from ImageJ analysis, from the packages Scikit‐learn (blue), AutoXAI4Omics (green), or AutoGluon (red). Black points represent mean values for each model−platform combination. Further model metrics and definitions of model labels are provided in Table 1.

**Table 1:** Model performance metrics across AI platforms in the seed species classification following cross‐validation.

Platform	Model	Label	Precision		Recall		Macro‐F1	
			Mean	SD	Mean	SD	Mean	SD
PyTorch	ResNet‐50 (Cellpose)	RN50_CP	0.990	0.010	0.987	0.010	0.989	0.008
AutoGluon	Neural Net Fast AI	NNFAI	0.972	0.016	0.969	0.012	0.970	0.012
AutoGluon	Weighted ensemble	Ensemble	0.971	0.016	0.969	0.011	0.970	0.012
AutoXAI4Omics	XGBoost	XGB	0.971	0.017	0.962	0.014	0.966	0.014
AutoXAI4Omics	Light gradient boosting	LGBM	0.969	0.020	0.959	0.015	0.963	0.015
Scikit‐learn	HistBoost	Hist	0.961	0.019	0.961	0.012	0.961	0.015
Scikit‐learn	Multilayer Perceptron	MLP	0.960	0.019	0.960	0.014	0.960	0.016
Scikit‐learn	XGBoost	XGB	0.958	0.021	0.958	0.013	0.958	0.017
Scikit‐learn	Gradient boosting	GB	0.956	0.022	0.956	0.014	0.956	0.017
AutoXAI4Omics	Multilayer Perceptron	MLP	0.954	0.024	0.957	0.017	0.955	0.018
AutoXAI4Omics	Random forest	RF	0.946	0.028	0.939	0.023	0.942	0.022
Scikit‐learn	Random forest	RF	0.946	0.028	0.939	0.023	0.942	0.022
AutoXAI4Omics	Gradient boosting	GB	0.940	0.028	0.942	0.014	0.941	0.021
AutoGluon	Extra‐Trees Gini	ETGini	0.933	0.033	0.925	0.030	0.929	0.029
AutoXAI4Omics	Auto‐XGBoost	AutoXGB	0.899	0.042	0.899	0.037	0.899	0.038
Scikit‐learn	*k*‐Nearest Neighbors	k‐NN	0.898	0.046	0.900	0.040	0.899	0.042
AutoXAI4Omics	*k*‐Nearest Neighbors	k‐NN	0.899	0.046	0.897	0.038	0.898	0.038
PyTorch	ResNet‐50 (ImageJ)	RN50_IJ	0.880	0.109	0.908	0.063	0.893	0.086
Scikit‐learn	AdaBoost	Ada	0.786	0.119	0.772	0.096	0.770	0.075
AutoGluon	*k*‐NN distance	knn_dist	0.208	0.114	0.194	0.127	0.198	0.115
AutoGluon	*k*‐NN uniform	knn_unif	0.179	0.091	0.170	0.135	0.168	0.112

We additionally performed a supplier‐held‐out evaluation to directly quantify provenance generalization when a supplier is absent from model training (Appendix S3). Overall, the strongest tabular‐feature models retained a high macro‐F1 score under supplier exclusion, and the relative ranking of models was consistent with the main pooled evaluation; the macro‐F1 score remained in a high range for the best‐performing models (e.g., macro‐F1 score = 0.93–0.94 for HistGradientBoosting and MLP, and 0.92 for random forest). This indicated that model performance was not driven by supplier overlap between training and evaluation data.

### Comparison of neural network classification models from image inputs

To train and evaluate a CNN for this classification task, we first generated individual seed images from the scanned images and their corresponding masks from the 10 wildflower species. We compared two methods for segmenting the scanned images and generating masks: a non‐AI pipeline built with the OpenCV v2 Python library (Bradski, 2000) and a neural network image segmentation model (Cellpose v4). The resulting datasets were comparable in size (35,310 and 35,373 individual seed images, respectively). When we overlaid the masks on the original images (Figure 1E, F compared to Figure 1H, I), it was clear that the Cellpose‐generated masks were more accurate and precise than those generated by OpenCV. However, the processing time required by Cellpose was significantly longer than the non‐AI OpenCV pipeline (~30 s/scan versus ~300 s/scan).

We initially compared three CNNs (ResNet‐50, InceptionV3, and MobileNetV3) on 700 images per class from the OpenCV‐generated dataset. After 100 epochs, the macro‐F1 score was 0.79 for MobileNetV3, 0.81 for InceptionV3, and 0.84 with ResNet‐50. We decided to proceed with ResNet‐50 for further optimization.

We optimized the pipeline by improving input transformations to better align with the model's expectations, augmenting training to prevent overfitting, balancing class weights during training, progressively tuning the model's deeper layers, and using both image datasets fully (over 34,000 instances in each). After 100 epochs, the macro‐F1 scores obtained by ResNet‐50 in the OpenCV‐generated (Appendix S5A) and Cellpose‐generated datasets (Appendix S5B) were 0.89 and 0.99, respectively. With the OpenCV‐generated dataset, the ResNet‐50 model exhibited slower convergence and higher loss, with moderate accuracy and F1 score stability, suggesting underfitting or data noise. In contrast, the model showed rapid loss reduction and consistently higher accuracy and F1 score with the Cellpose‐generated dataset, indicating improved generalization and more effective optimization. These results demonstrate that dataset differences alone substantially influence network learning trajectories and overall predictive reliability relative to model optimization.

### Open‐set classification

Under realistic conditions, some taxa may be absent from the labeled training data but may be present in market mixes, leading to open‐set classification. To assess the capacity of the best models to handle unseen classes (i.e., unseen species), we generated both tabulated and seed image data from additional common wildflower species: *Centaurea cyanus* L., *Echium vulgare* L., *Glebionis segetum* (L.) Fourr., *Medicago lupulina* L., *Plantago lanceolata* L., *Primula veris* L., *Rhinanthus minor* L., and *Silene dioica* (L.) Clairv. In this scenario, models trained with the full training set with the known species were later evaluated with mock seed mixes consisting of an equal number of instances of known species (classes) from the balanced evaluation set (i.e., not used in training) and the same number of instances from the new unseen species. In the absence of calibration or thresholding, all these models aimed to classify each unseen instance in the mock seed mixes into one of the known classes, resulting in a reduction of F1 score in all models and classes (Figure 5).

**Figure 5:**
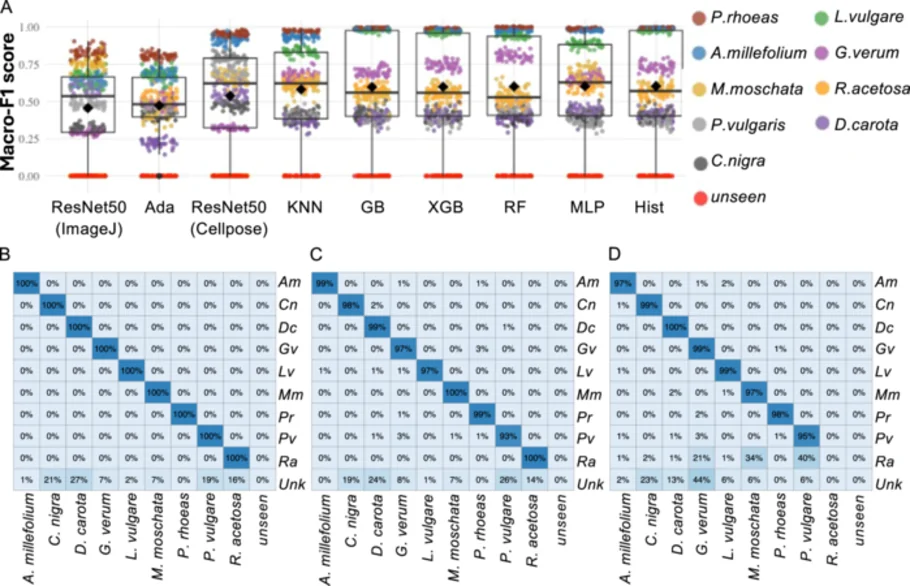
Performance in open‐set classification in the absence of thresholding. (A) Macro‐F1 scores for open‐set classification using default decision thresholds for model prediction on mock seed mixes containing 50% known species and 50% unseen species. (B–D) Confusion matrices comparing the observed and predicted species for nine known species in the training set and the unseen class of species added during validation for open‐set classification using default decision thresholds for three selected models. Definitions of model labels are provided in Table 1.

In order to improve the performance at handling unseen classes, we evaluated the effect of incorporating a decision threshold variable, so the class with the maximum probability was taken as the predicted species only if its confidence exceeded the predefined decision threshold; otherwise, the image was categorized as “unclassified.” To decide on the best threshold value, the mock seed mixes datasets were used to estimate the coverage, precision, recall, and F1 score for each model at 0.05 threshold increments and generate threshold performance plots (Appendix S6). As the threshold increases over the default value of 0.5, the recall starts decreasing and precision starts increasing (Appendix S6). A good balance or trade‐off between these two metrics can be obtained by selecting the probability threshold that maximizes the F1 score. Boosting algorithms such as XGBoost (Appendix S6A) operate at fluctuating thresholds close to 1, whereas random forest (Appendix S6B) is consistently optimal at 0.8. The optimum thresholds were AdaBoost = 0.2, GradientBoosting = 0.95, *k*‐nearest neighbors (*k*‐NN) = 0.9, HistGradientBoosting = 0.95, MLP = 0.95, RandomForest = 0.8, and XGBoost = 0.95; the optimum threshold of ResNet‐50 was the largest evaluated (0.95).

Applying a decision threshold significantly improved the open‐set performance for all models with the mock seed mixes for all species, with the random forest classifier and the FastAI neural network overperforming the other models (Figure 6, Table 2). Comparing the confusion matrices with and without thresholding for the same model illustrated the effect of calibration in transitioning from closed to open‐set classification (Figure 5 compared to Figure 6).

**Figure 6:**
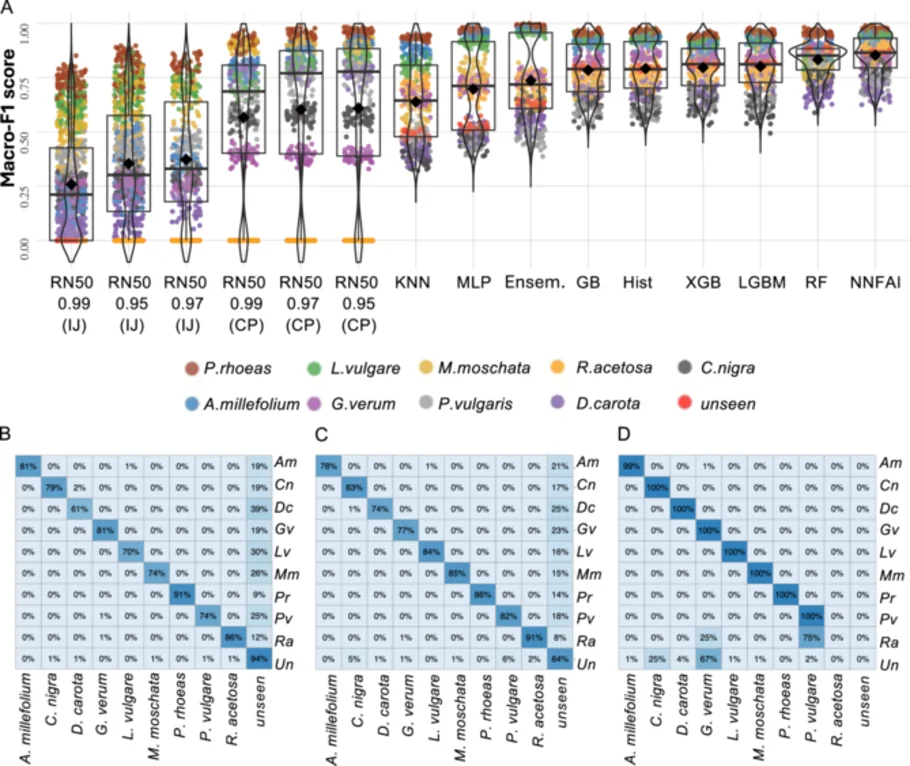
Performance in open‐set classification incorporating a decision threshold. (A) Macro‐F1 scores for open‐set classification using optimal decision thresholds for model prediction on mock seed mixes containing 50% known species and 50% unseen species. Black diamonds represent macro‐F1 values. (B–D) Confusion matrices comparing the observed and predicted species for nine known species in the training set and the unseen class of species added during validation for open‐set classification using default decision thresholds for three selected models. Definitions of model labels are provided in Table 1.

**Table 2:** Open‐set model evaluation using thresholded models.^a^

Platform	Model	Label	Macro‐F1		Precision		Recall	
			Mean	SD	Mean	SD	Mean	SD
AutoGluon	*k*‐NN uniform weighted	knn_unif	0.074	0.197	0.168	0.304	0.103	0.295
Scikit‐learn	AdaBoost	Ada	0.120	0.209	0.292	0.409	0.127	0.282
AutoGluon	*k*‐Nearest Neighbors	k‐NN	0.673	0.182	0.630	0.255	0.838	0.166
Scikit‐learn	*k*‐Nearest Neighbors	k‐NN	0.673	0.182	0.630	0.255	0.838	0.166
Scikit‐learn	Multilayer Perceptron	MLP	0.708	0.195	0.663	0.260	0.877	0.171
AutoGluon	Weighted ensemble	Ensemble	0.747	0.176	0.691	0.253	0.916	0.149
AutoGluon	Gradient boosting	GB	0.799	0.113	0.772	0.190	0.860	0.068
Scikit‐learn	XGBoost	XGB	0.807	0.099	0.802	0.173	0.838	0.073
AutoGluon	HistBoost	Hist	0.807	0.116	0.775	0.195	0.881	0.072
AutoGluon	Light gradient boosting	LGBM	0.812	0.109	0.790	0.185	0.867	0.064
AutoGluon	Neural Net Fast AI	NNFAI	0.855	0.092	0.848	0.133	0.869	0.065
AutoGluon	*k*‐NN distance weighted	knn_dist	0.882	0.033	0.965	0.066	0.819	0.070
Scikit‐learn	Random forest	RF	0.908	0.067	0.882	0.116	0.945	0.043
PyTorch	resnet_cellpose_095thr	resnet_cellpose_095thr	0.601	0.380	0.671	0.346	0.609	0.346
PyTorch	resnet_cellpose_097thr	resnet_cellpose_097thr	0.619	0.386	0.635	0.333	0.602	0.340
PyTorch	resnet_cellpose_099thr	resnet_cellpose_099thr	0.647	0.396	0.543	0.300	0.567	0.321
PyTorch	resnet_imageJ_095thr	resnet_imageJ_095thr	0.554	0.405	0.321	0.241	0.353	0.270
PyTorch	resnet_imageJ_097thr	resnet_imageJ_097thr	0.555	0.403	0.343	0.240	0.372	0.272
PyTorch	resnet_imageJ_099thr	resnet_imageJ_099thr	0.496	0.431	0.213	0.202	0.258	0.249

a Performance metrics represent macro averages based on evaluation datasets consisting of 50% unseen classes.

## DISCUSSION

Previous seed classifiers focused on distinguishing among economically important crop varieties and commercial hybrids (Ahmad et al., 1999; Khatri et al., 2022; Madhavan et al., 2023) and identifying contaminants in the form of weed seeds (Granitto et al., 2002; Luo et al., 2023). At the same time, user‐friendly applications for seed identification that incorporate generative AI have been released for mobile devices (e.g., Seed Identifier [Indie Mobile Apps] and Nuts & Seeds [MSDLDevelopers]). The former can identify the species based on the seed (one species) but does not handle frequency or mixtures; the second is a database of seed images in which the user can locate the correct species. These commercial applications are closed source, so we do not know the species they are trained on, but they seem to rely on large language models (LLMs) trained on online images. These analyses result in a general description that may be accurate but lacks the granularity and labeled detail that can adequately substitute for a human expert.

### Model evaluation

We compared two segmentation pipelines, a non‐AI segmentation pipeline built on ImageJ and an AI‐based pipeline built on the Cellpose v4 CNN. We also compared species classification based on either tabulated values or single seed images. This allowed us to directly compare tabulated‐feature machine learning and deep learning models with a state‐of‐the‐art CNN. Our evaluation showed the strongest performance in closed‐set classification for ResNet‐50 when trained using Cellpose‐derived masks, which yielded a macro‐F1 score of 0.99 after fine‐tuning and data augmentation. These results demonstrate that segmentation quality is a critical determinant of deep learning performance and that inexpensive scanner images can support high‐accuracy CNN models when paired with robust masking. Therefore, segmentation should be regarded as a fundamental design decision rather than just a basic preprocessing step.

We also compared tools with automated optimization (AutoXAI4Omics and AutoGluon) to basic grid searching in Scikit‐learn (Pedregosa et al., 2011; Erickson et al., 2020; Strudwick et al., 2025). Overall, automatic parameter tuning did not provide significant performance gains, but it did streamline the coding process. This benefit was offset by reduced flexibility compared to using Scikit‐learn directly.

The AutoGluon platform featured a wider array of intricate ensemble and deep learning classifiers, with two of its “black box” deep learning models, weighted ensemble (WE) and Neural Net FastAI (NNFAI), attaining F1 scores of 0.97 in closed‐set classification, just below the F1 score achieved using ResNet‐50 with Cellpose‐segmentation. This weighted ensemble model combines multiple base models (e.g., Light Gradient‐Boosting Machine [LightGBM], CatBoost, Neural Nets, *k*‐NN), whereas NNFAI is a feed‐forward neural network designed for tabular data and built on the FastAI deep learning toolbox. In contrast, traditional machine learning models like random forests and various gradient boosting methods (XGBoost, HGB, LightGBM) achieved comparable F1 scores (ranging from 0.94 to 0.96) in closed‐set classification, while requiring less computational resources in terms of time and memory and offering greater flexibility.

The near‐perfect performance of ResNet‐50 on the Cellpose dataset highlights a comprehensive solution, but it comes at the cost of additional computational burden and the need for large volumes of well‐segmented images, which also increase computational demands when using a CNN for segmentation. This supports the idea that deep learning may not always provide the most practical solution, as random forest and XGBoost from tabulated data offer metrics that are not significantly behind, despite requiring far fewer computational resources. Therefore, model choice should be framed as a cost‐accuracy trade‐off rather than as a single “best” approach for every use case.

Classification performance was also persistently related to species, likely due to different levels of intraspecific variation and consistent divergence from average feature values among species in the dataset. The wild carrot (*Daucus carota* L.), one of the most difficult seeds to classify, is brittle and morphologically diverse, which underscores the greater variance in morphological features observed in the PCA and LDA analyses. Species with glossy or translucent seeds, like *Centaurea nigra* L. and *Achillea millefolium* L., were occasionally difficult to segment, introducing small errors in the shape and size values extracted. These sensitivities were also visible in the CNN models, where misclassification rates were driven by differences in segmentation rather than classification (i.e., higher when using ImageJ‐generated masks), reinforcing the importance of robust image preprocessing across both machine learning and deep learning approaches. This indicates that certain species that are difficult to identify might need specialized data augmentation, enhanced pre‐processing, or species‐specific classification methods.

### Open‐set classification

Our results demonstrate that ensemble machine learning models designed for closed‐set applications can be effectively adapted for open‐set tasks using thresholding. Without intervention, closed‐set classifiers assume that any new input belongs to one of the known classes, resulting in classification within those existing categories. By applying thresholding and introducing an additional “unseen” class, we can ensure that instances are only classified within the known species if they surpass a specified confidence threshold. In practice, our tool must accommodate this variation and disregard unseen classes (new species), i.e., the challenge of unseen classes during training (open‐set classification). Commercial native seed mixes typically include seeds from various species, such as grasses and wildflowers, at varying frequencies based on supply factors (year, season, local abundance) and demand factors (target habitat, indicator species). This reality establishes open‐set classification as a fundamental requirement rather than an exception.

Our results show that the effectiveness of a fixed threshold depends on the model architecture and may not be possible in some models. Random forest achieved higher F1 scores due to a stable probability threshold from averaging trees. Random forest, which is based on tree averaging, is well suited for fixed thresholds because of its stable decision gradient. In contrast, gradient descent models like XGBoost struggle with fixed thresholds because of steep exponential loss functions, which yield a less determinate decision boundary. Therefore, open‐set conditions should focus on models that produce “stable” probabilities or incorporate explicit calibration.

The inclusion of deep learning models enabled us to extend these findings to CNN‐based image classification. ResNet‐50 exhibited highly variable behavior in open‐set classification, and even at a 0.99 threshold, most “unseen” instances were compelled to be classified into one of the known categories (Figure 6D). We also observed significant performance differences between classes, where some classes that were easily classified in a closed set (Figure 5D), such as *Rumex acetosa* L., failed during classification when unseen classes were introduced (Figure 6D). Because the NNFA neural network could handle open‐set classifications from tabulated data, this might be specific to open‐set classification from individual seed images, which may require different strategies (e.g., temperature scaling).

### Concluding remarks

This study presented methods for classifying wildflower seeds using image analysis and AI. The optimal method depends on the balance between accuracy and computational time, the use of AutoML tools, and whether the classification involves a closed set of species. If the focus is on absolute metrics without regard for computational cost, neural networks for segmentation (CellPose) and classification (ResNet‐50) proved to be the best options. However, generating tabulated features was an order of magnitude faster than deep learning segmentation. In closed‐set classification, the complex models from AutoGluon were the most accurate, but four models from vanilla Scikit‐learn performed similarly. In open‐set classification, random forests clearly excelled because of their transparent use of calibration and thresholds to assign unseen species to the unclassified class. Our findings indicate that traditional machine learning models, such as random forests and gradient boosting, achieve high classification accuracy with lower computational demands than deep learning methods. Nonetheless, our extended analyses also demonstrated that deep learning (ResNet‐50) can achieve almost perfect accuracy when supplied with high‐quality segmentation (e.g., Cellpose), offering a pathway towards fully automated image‐to‐classification pipelines at a higher computational cost. Furthermore, we show that open‐set classification can be effectively managed using probability thresholding, enhancing the tool's capacity to handle unseen species in real‐world scenarios. Future research may explore new strategies for robust open‐set classification to produce results more comparable to the comprehensive results obtained for closed‐set classification. We also plan to test the effect of seed aging on classification, so the approaches can be utilized in seedbank collections. Overall, our findings offer practical guidance for choosing a seed identification method that balances accuracy, computational cost, and robustness in real‐world conditions.

## AUTHOR CONTRIBUTIONS

J.A. and J.J.D.V. designed the research. J.A., E.B., R.H., B.W., and F.S. conducted the experimental work, coding, and analyzed the data. A.H. and J.J.D.V. provided supervision and obtained funding. J.A. and J.J.D.V. prepared the manuscript. All the authors approved the final version of the manuscript.

## Supporting information


**Appendix S1:** Scientific and common names of the 10 wildflower species analyzed, with the total number of seeds scanned and incorporated into our dataset via image analysis. Each species consists of stock derived from five independent sources.

**Appendix S2:** Sixty‐four image‐based features extracted using the generic_background_analyzer plug‐in.

**Appendix S3:** Supplier‐held‐out evaluation (provenance generalization): per‐class precision, recall, F1 score, and macro‐F1 score for each model per supplier, which was held out from training.

**Appendix S4:** The top five features by loading for the first three principal components based on the full unbalanced dataset.

**Appendix S5:** Training and validation loss curves across 100 epochs for the ResNet‐50 CNN classification using either the non‐AI ImageJ/OpenCV‐segmented dataset (A) or the AI Cellpose‐segmented dataset (B).

**Appendix S6:** Threshold‐tuning curves for handling unseen species in open‐set classification. The models’ performance plots indicate averaged efficiency metrics (F1, precision, recall, coverage) at 0.05 threshold increments. Boosting algorithms such as XGBoost (A) operate at fluctuating thresholds very close to 1, whereas random forest (B) is consistently optimal at 0.8. (C–F) ResNet‐50 evaluated using ImageJ‐derived (C, E) or Cellpose‐derived (D, F) datasets reflect that when thresholds were increased, the recall values decreased but the precision values increased. the optimal threshold was selected when these threshold values were the highest. However, this also resulted in a macro‐F1 score that was lower than in other methods because of the greater variation between classes (E, F).

## Data Availability

The ImageJ macro and plug‐in, all the Python scripts used to segment and extract the images and to train and evaluate the models, submission scripts and config files for the AutoML tools, and all the trained models generated are available under the open MIT license at https://github.com/DeVegaGroup/SeedClassifier.
