# Supplementary material for: Integrating machine learning, deep learning, and image analysis for seed species classification
Source: Appl Plant Sci. 2026 Jul 23;14(5):e70072. doi: 10.1002/aps3.70072 (PMC13720345; doi:10.1002/aps3.70072)

DOI 10.1002/aps3.70072

APPENDIX S5. Training and validation loss curves across 100 epochs for the ResNet-50 CNN classification using either the non-AI ImageJ/OpenCV-segmented dataset (A) or the AI Cellpose-segmented dataset (B).

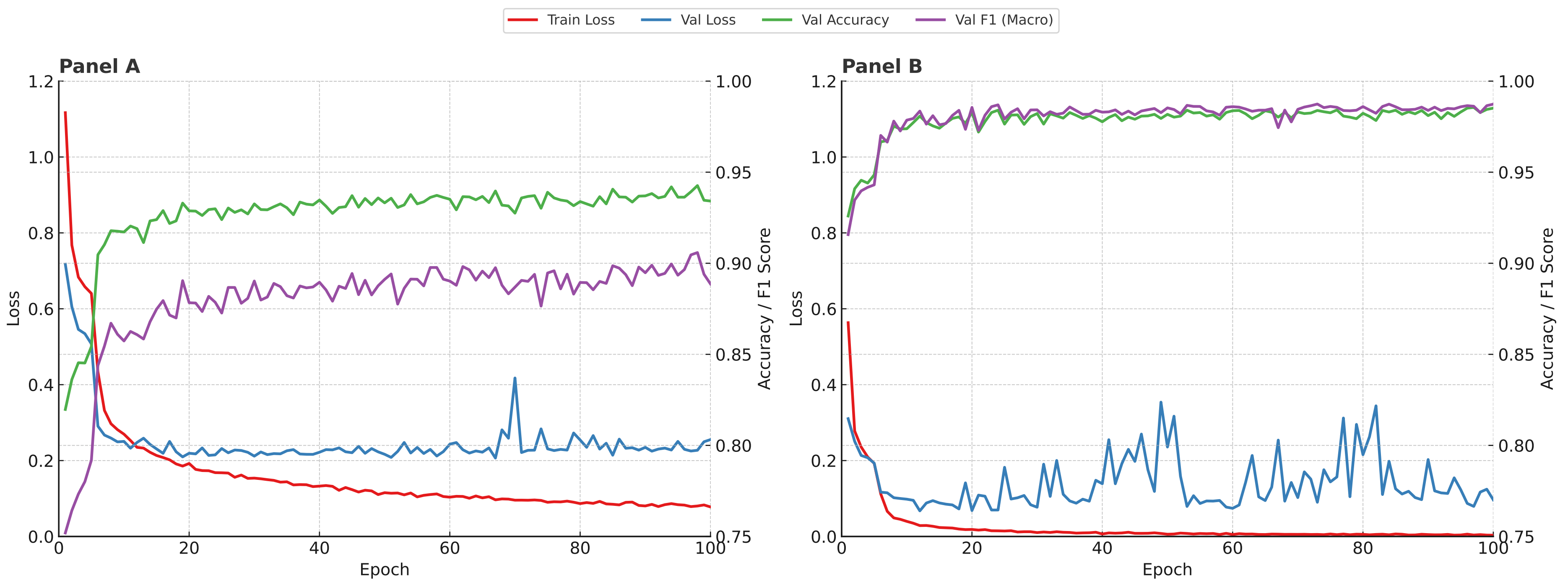

Supplement: Supplementary file 1 [file APS3-14-e70072-s005.pdf]
