# Supplementary material for: Integrating machine learning, deep learning, and image analysis for seed species classification
Source: Appl Plant Sci. 2026 Jul 23;14(5):e70072. doi: 10.1002/aps3.70072 (PMC13720345; doi:10.1002/aps3.70072)

DOI 10.1002/aps3.70072

APPENDIX S6. Threshold-tuning curves for handling unseen species in open-set classification. The models' performance plots indicate averaged efficiency metrics (F1, precision, recall, coverage) at 0.05 threshold increments. Boosting algorithms such as XGBoost (A) operate at fluctuating thresholds very close to 1, whereas random forest (B) is consistently optimal at 0.8. (C–F) ResNet-50 evaluated using ImageJ-derived (C, E) or Cellpose-derived (D, F) datasets reflect that when thresholds were increased, the recall values decreased but the precision values increased. the optimal threshold was selected when these threshold values were the highest. However, this also resulted in a macro-F1 score that was lower than in other methods because of the greater variation between classes (E, F).

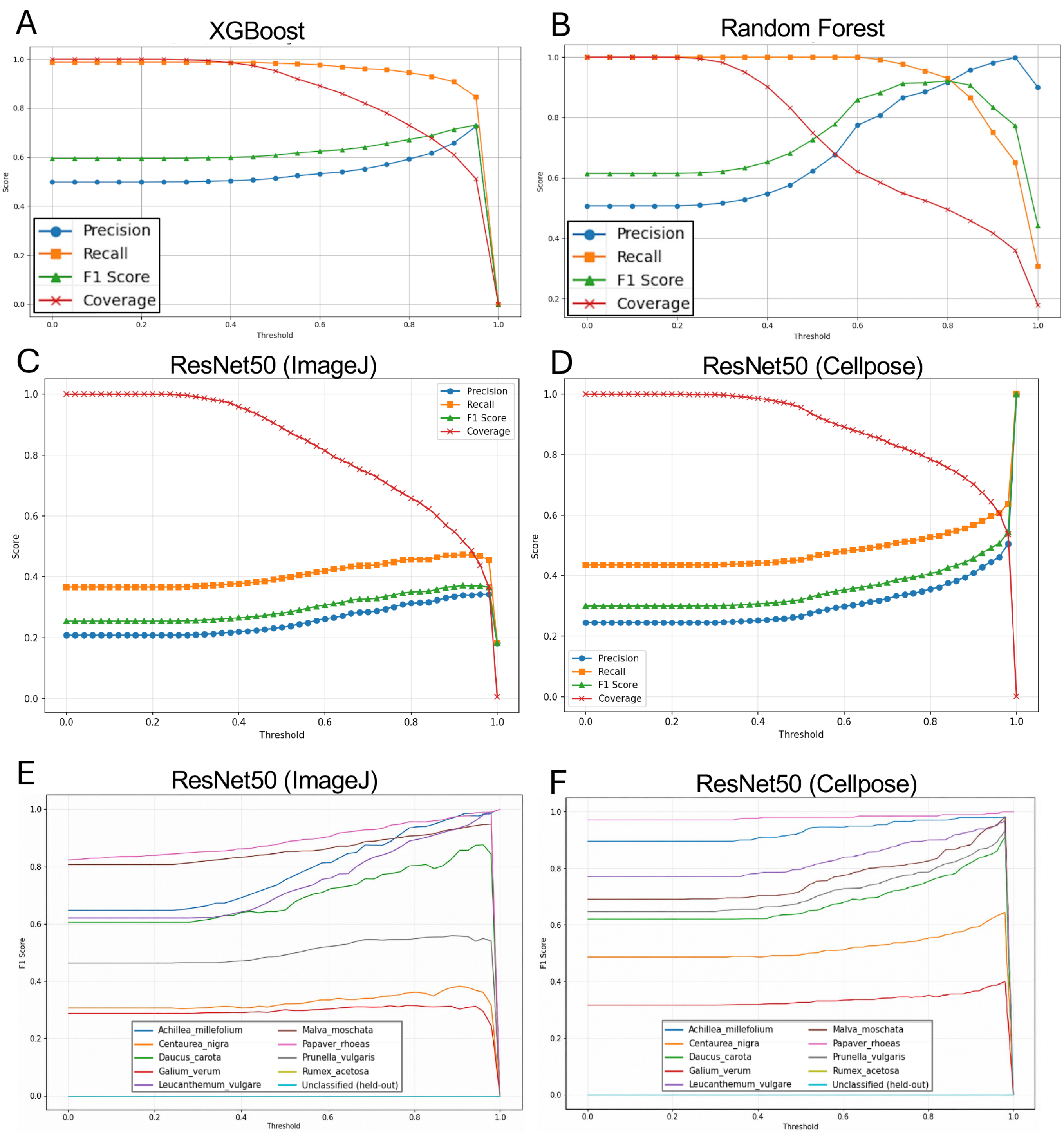

Supplement: Supplementary file 2 [file APS3-14-e70072-s006.pdf]
