# Supplementary material for: Integrating machine learning, deep learning, and image analysis for seed species classification
Source: Appl Plant Sci. 2026 Jul 23;14(5):e70072. doi: 10.1002/aps3.70072 (PMC13720345; doi:10.1002/aps3.70072)
Supplement: Supplementary file 3 [file APS3-14-e70072-s001.docx]

**Appendix S2.** Sixty-four image-based features extracted using the generic_background_analyzer plug-in.

| **Feature** | **Description** | **Type** |
| --- | --- | --- |
| Area | Area (in pixels) | Morphology |
| Mean | Average gray value | Texture |
| Mode | Mode of gray values | Texture |
| Min | Minimum gray value | Texture |
| Max | Maximum gray value | Texture |
| Median | Median of gray values | Texture |
| Perimeter | Length of outside edge | Morphology |
| X.Feret | Length at longest point | Morphology |
| X.Breadth | Widest perpendicular to Feret | Morphology |
| X.AspRatio | Feret/breadth | Morphology |
| X.RFactor | ConvexArea/(Feret x 𝜋) | Morphology |
| X.ConvexArea | Area of convex polygon drawn between external points | Morphology |
| X.ConvexPerimeter | Perimeter of convex polygon | Morphology |
| X.ArEquivD | Diameter of the circle with equivalent area of the region, defined as sqrt(4*Area/π) | Morphology |
| X.PerEquivD | Diameter of the circle having the same perimeter of the region | Morphology |
| X.MinR | Radius of inscribed circle | Morphology |
| X.MaxR | Radius of enclosing circle | Morphology |
| X.avgR | Average length of radius from center of mass | Morphology |
| X.VarianceR | Variance of radii | Morphology |
| X.EquivEllAr | Area of ellipse having Feret and Breadth as axes | Morphology |
| X.ModRatio | (2xMinR)/Feret | Morphology |
| X.HaralickRatio | GLCM second-order statistics (Energy, Contrast, Correlation, Homogeneity) | Texture |
| X.ThinnessR | *Perimeter^2^ / Area* | Morphology |
| X.Roundness | $4 \times Area/(\pi\times{Feret}^{2})$ | Morphology |
| X.Compactness | $\sqrt{\frac{4}{\pi} \times Area/Feret}$ | Morphology |
| X.Solidity | Area/ConvexArea | Morphology |
| X.Convexity | *ConvexPerimeter/Perimeter* | Morphology |
| X.Concavity | ConvexArea – Area | Morphology |
| X.ArBBox | Area of bounding box containing the region | Morphology |
| X.Rectang | Area/ArBBox | Morphology |
| X.Sphericity | MinR/MaxR | Morphology |
| X.Elongation | ${Perimeter}^{2}/(4 \times\pi\times Area)$ | Morphology |
| X.Bending.Energy | Sum of squared curvature along perimeter | Morphology |
| X.Jaggedness | $(2 \times\sqrt{\pi\times Area})/Perimeter$ | Morphology |
| X.Circularity | $2\times\pi\times Area/{Perimeter}^{2}$ | Morphology |
| X.Endocarp | Number of pixels | Morphology |
| Distance.CM.FB | Distance from center of mass to farthest boundary | Morphology |
| StD | Intensity standard deviation as contrast measure | Texture |
| Skewness | Measure of the symmetry of the gray-level histogram around the average value | Texture |
| Kurtosis | Measure of the *tailedness* of the gray-level histogram | Texture |
| Intensity.sum | Sum of gray values | Texture |
| Uniformity | Maximum when all the gray levels in the histogram are equal | Texture |
| Entropy | Variability of gray-level distribution | Texture |
| Smoothness.R | Measure of smoothness | Texture |
| GLCM.Contrast | Measure of variability in gray tone values | Texture |
| GLCM.Correlation | Spatial correlation in gray tone values | Texture |
| GLCM.Energy | GLCM variability of gray-level distribution | Texture |
| GLCM.Homogeneity | Proportion of spatial transitions in gray tone | Texture |
| Mean.Red (MR) | Average red channel value | Color |
| Sqrt.Mean.Red | Square root of mean red channel values | Color |
| StD.Red | Standard deviation of red channel values | Color |
| Mean.Green (MG) | Average green channel value | Color |
| Sqrt.Mean.Green | Square root of mean green channel value | Color |
| StD.Green | Standard deviation of green channel values | Color |
| Mean.Blue (MB) | Average of blue channel values | Color |
| Sqrt.Mean.Blue | Square root of the mean value for blue channel | Color |
| StD.Blue | Standard deviation of blue channel values | Color |
| Mean.RGB | $\frac{MR+MG+MB}{3}$ | Color |
| Mean.H | Average tone of hue channel | Color |
| StD.H | Standard deviation of hue channel values | Color |
| Mean.S | Average tone of saturation channel | Color |
| StD.S | Standard deviation of saturation channel values | Color |
| Mean.V | Average tone of value channel | Color |
| StD.V | Standard deviation of value channel values | Color |
