# Supplementary material for: Integrating machine learning, deep learning, and image analysis for seed species classification
Source: Appl Plant Sci. 2026 Jul 23;14(5):e70072. doi: 10.1002/aps3.70072 (PMC13720345; doi:10.1002/aps3.70072)
Supplement: Supplementary file 4 [file APS3-14-e70072-s002.docx]

**Appendix S4.** The top five features by loading for the first three principal components based on the full unbalanced dataset.

| **Principal component** | **Feature** | **Type** | **Loading value** |
| --- | --- | --- | --- |
| PC1 | MaxR | Morphological | 0.215 |
| PC1 | Feret | Morphological | 0.215 |
| PC1 | Endocarp | Morphological | -0.213 |
| PC1 | PerEquivD | Morphological | 0.213 |
| PC1 | Perimeter | Morphological | 0.2125 |
| PC2 | Mean.Green | Color | 0.249 |
| PC2 | Mean.RGB | Color | 0.247 |
| PC2 | Sqrt.Mean.Green | Color | 0.247 |
| PC2 | Mean.Red | Color | 0.246 |
| PC2 | Mean.V | Color | 0.245 |
| PC3 | Entropy | Texture | 0.3720 |
| PC3 | StD | Texture | 0.3628 |
| PC3 | Uniformity | Texture | -0.3539 |
| PC3 | Min | Texture | -0.3328 |
| PC3 | Smoothness.R | Texture | 0.3311 |
