# Supplementary material for: Integrating machine learning, deep learning, and image analysis for seed species classification
Source: Appl Plant Sci. 2026 Jul 23;14(5):e70072. doi: 10.1002/aps3.70072 (PMC13720345; doi:10.1002/aps3.70072)
Supplement: Supplementary file 5 [file APS3-14-e70072-s004.docx]

**Appendix S1.** Scientific and common names of the 10 wildflower species analyzed, with the total number of seeds scanned and incorporated into our dataset via image analysis. Each species consists of stock derived from five independent sources.

| **Species** | **Common name** | **Rows/seeds** |
| --- | --- | --- |
| *Achillea millefolium* | Yarrow | 21834 |
| *Centaurea nigra* | Knapweed | 9590 |
| *Daucus carota* | Wild carrot | 11540 |
| *Galium verum* | Lady’s bedstraw | 19685 |
| *Knautia arvensis* | Field scabious | 662 |
| *Leucanthemum vulgare* | Oxeye daisy | 15262 |
| *Malva moschata* | Musk mallow | 6103 |
| *Papaver rhoeas* | Corn poppy | 38108 |
| *Prunella vulgaris* | Selfheal | 10887 |
| *Rumex acetosa* | Common sorrel | 12100 |
